# The LKB1–AMPK Signaling Axis Modulates Ferroptosis in Fibroblast-Like Synoviocytes Derived from Rheumatoid Arthritis

**DOI:** 10.3390/biomedicines13020321

**Published:** 2025-01-30

**Authors:** Ha-Reum Lee, Su-Jin Yoo, Jinhyun Kim, Seong Wook Kang

**Affiliations:** 1 Research Institute for Medical Sciences, School of Medicine, Chungnam National University, Daejeon 35015, Republic of Korea; hareum_lee@cnu.ac.kr (H.-R.L.); sujin428@cnuh.co.kr (S.-J.Y.); jkim@cnuh.co.kr (J.K.); 2 Division of Rheumatology, Department of Internal Medicine, Chungnam National University Hospital, Daejeon 35015, Republic of Korea

**Keywords:** rheumatoid arthritis, fibroblast-like synoviocytes, ferroptosis, metabolism

## Abstract

Background/Objectives: Ferroptosis is a type of regulated cell death that involves iron-dependent accumulation of lipid peroxides. Because fibroblast-like synoviocytes (FLSs) in patients with rheumatoid arthritis (RA) have a hyperplastic and inflammatory phenotype, selective induction of FLS cell death is considered a potential treatment strategy for RA. Liver kinase B1 (LKB1)-activated AMP-activated protein kinase (AMPK) signaling regulates the inflammation and migration of RA FLSs, contributing to RA pathogenesis. Here, we aimed to determine the effect of LKB1 knockdown on the ferroptosis pathway in RA FLSs. Methods: Synovial tissues from patients with RA (*n* = 5) were transfected with siRNA targeting LKB1. Cell viability was evaluated via 3-[4,5-dimethylthiazol-2-yl]-2,5 diphenyl tetrazolium bromide (MTT) assay and Annexin V/7-aminoactinomycin D (7-AAD) staining. Ferroptosis was assessed using boron-dipyrromethene (BODIPY) lipid probes, a ferrous ion detection kit, and a glutathione detection assay. Expression of hallmarks of various cell death pathways was analyzed using western blot. Results: RA FLS cell death significantly increased after transfection with LKB1 siRNA (*p* < 0.01). Lipid peroxidation was upregulated and the expression levels of glutathione peroxidase 4 (GPX4) and solute carrier family 7 member 11 (SLC7A11) were suppressed in LKB1-deficient cells. Additionally, LKB1 inhibition made RA FLSs highly sensitive to ferroptosis. When RA FLSs were incubated with an activator of AMPK, LKB1 knockdown-mediated inhibition was restored through upregulated expression of GPX4 and SLC7A11. Conclusions: these findings suggest that LKB1–AMPK signaling is essential to protect RA FLSs against ferroptosis.

## 1. Introduction

Rheumatoid arthritis (RA) is an autoimmune inflammatory disease with abnormal proliferation and inflammation of synovial tissue, which lead to joint destruction [1,2]. Fibroblast-like synoviocytes (FLSs) reside in the intimal lining of the synovium and are responsible for RA progression [3]. In RA, FLSs present antigens to activated T cells and induce synovial inflammation due to excessive immune cell recruitment [4]. Furthermore, RA FLSs exhibit an overgrowth phenotype and resistance to programmed cell death pathways, leading to synovial hypertrophy [5]. Specific targeting of synovial proliferation is considered a potential strategy for RA treatment and has been evaluated in preclinical and clinical research [6].

Cell death is essential for the maintenance of homeostasis and the prevention of hyperproliferation in response to damage or infection [7]. There are several well-known cell death pathways, including caspase-3-dependent apoptosis [8], light chain 3 (LC3)A/B-mediated autophagy [9], gasdermin D-mediated pyroptosis [10], receptor-interacting protein kinase (RIPK)-dependent necroptosis [11], and iron-dependent phospholipid peroxidation–mediated ferroptosis [12], each with different characteristics of molecular regulation [13]. Ferroptosis was first identified as a type of programmed cell death characterized by oxidative membrane damage and overload of intracellular iron and lipid peroxides [14]. Because cells undergoing ferroptosis usually show necrosis-like morphological features such as plasma membrane swelling or rupture, ferroptosis is easily distinguished from conventional apoptosis [15].

Ferroptosis can be triggered by inhibition of the amino acid antiporter system Xc^−^ or the glutathione (GSH) peroxidase 4 (GPX4) antioxidant system [16]. System Xc^−^, composed of solute carrier family 7 member 11 (SLC7A11) and solute carrier family 3 member 2 (SLC3A2), regulates the exchange of cystine and glutamate in cell membranes [17]. GPX4 belongs to the GSH peroxidase family and eliminates excess reactive oxygen species (ROS) by circulating between oxidated and reduced states [18]. The Xc^−^–GSH–GPX4 axis is regarded as the critical signaling pathway for protection against oxidative stress and ferroptosis [19]. Excessive ROS react with polyunsaturated fatty acids in cellular membranes, leading to lipid peroxidation and cell death via ferroptosis [20]. Lipid peroxidation is a chemical process involving the oxidative degradation of lipids and the formation of lipid radicals, which subsequently interact with multiple cellular metabolic pathways [21]. Because the prerequisite for lipid peroxidation is excessive ROS- and iron-dependent lipid peroxidation in cellular membranes, cellular sensitivity to lipid radicals seems to be highly dependent on oxidative stress and cellular lipid/iron metabolism [22].

Liver kinase B1 (LKB1) is known as a tumor suppressor and protects cells from intracellular ROS and metabolic changes [23]. The LKB1-activated AMP-activated protein kinase (AMPK) axis modulates multiple cellular functions, including energy imbalance, metabolic reprogramming, and oxidative phosphorylation [24]. In a previous study, we reported that LKB1 knockdown regulated SLC7A11–nicotinamide adenine dinucleotide phosphate (NADPH) oxidase 4 (NOX4)–ROS signaling in RA FLSs [25]. Here, we examined the effect of LKB1 knockdown on the ferroptosis pathway and other types of programmed cell death in RA FLSs. Our findings show that loss of LKB1 reduces RA FLS survival mainly by upregulating ferroptosis. These effects were reversed through activation of AMPK signaling with the AMPK-specific activator A769662. These results suggest that LKB1–AMPK signaling plays a critical role in determining ferroptosis susceptibility in RA FLSs. Our findings revealed new evidence highlighting the role of LKB1-regulated ferroptosis in RA pathology.

## 2. Materials and Methods

### 2.1. Human Subjects and Ethics Statement

Synovial tissues were obtained from five female patients with RA who were undergoing synovectomy or joint replacement (Table 1). The diagnosis of RA was confirmed according to the American College of Rheumatology (ACR)/European League Against Rheumatism (EULAR) 2010 classification criteria [26] at the Rheumatology outpatient clinic of Chungnam National University Hospital (Daejeon, Republic of Korea), with patients diagnosed between 2019 and 2021 included in this study. Patients with inflammatory diseases such as septic arthritis, infectious diseases, knee surgery, sclerosis, cancer, or immune disorders were excluded from the study. The synovium was cleaned of fibrous tissues and fat and dissociated with 0.1% collagenase (Sigma-Aldrich, St. Louis, MO, USA) in Dulbecco’s modified Eagle’s medium (DMEM, Gibco, Thermo Fisher Scientific, Waltham, MA, USA) at 37 °C for 90 min. Isolated primary FLSs were identified by CD90 expression with a purity >95% and used for subsequent experiments after four to six passages. Cells were cultured in DMEM supplemented with 10% fetal bovine serum (Gibco) and antibiotic–antimycotic solution (including 25 mg/mL amphotericin B, 100 U/mL penicillin, and 100 mg/mL streptomycin) (Cat# LS203-01, Welgene, Gyeongsangbuk-do, Republic Korea) and maintained in a 5% CO_2_ incubator at 37 °C. This study was performed in line with the recommendations of the Declaration of Helsinki. Approval was granted by the Institutional Review Board of Chungnam National University Hospital (CNUH 2019-12-068). Written informed consent was obtained from each patient.

### 2.2. siRNA Transfection

A specific siRNA duplex targeting LKB1 was purchased from Santa Cruz Biotechnology (Cat# sc-35816, Dallas, TX, USA). A control siRNA (Cat# sc-37007, Santa Cruz Biotechnology, Dallas, TX, USA) was used as a negative control. Lipofectamine 3000 (Invitrogen, Carlsbad, CA, USA) was employed to transfect FLSs following the manufacturer’s instructions. After 24 h of transfection, the endogenous expression of the target protein was evaluated. Images were observed under a light microscope (Olympus, Tokyo, Japan) equipped with a 0.55 numerical aperture dry objective at a magnification of 100× or 40× (scale bar, 200 µm).

### 2.3. MTT Assay

Cell viability was measured using a 3-[4,5-dimethylthiazol-2-yl]-2,5 diphenyl tetrazolium bromide (MTT) assay kit (Cat# 11465007001, Roche, Basel, Switzerland) according to the manufacturer’s instructions. In brief, cells were seeded in a 96-well plate and incubated with 5 mg/mL of MTT solution. Four hours later, solubilization buffer was added. After 18 h, solubilized formazan dye was quantified using a Sunrise absorbance reader at 570 nm (Tecan, Männedorf, Switzerland). The measured absorbance value directly indicates the number of viable cells.

### 2.4. Flow Cytometric Analysis

Cell death was assessed using fluorescein isothiocyanate (FITC)-conjugated Annexin V (BD Biosciences, Franklin Lakes, NJ, USA) and 7-aminoactinomycin D (7-AAD; BD Biosciences). To detect ROS levels, cells were stained with MitoSOX™ Red mitochondrial superoxide indicator (Invitrogen, Carlsbad, CA, USA; 500 nM) according to the manufacturer’s instructions. Lipid peroxidation was detected using boron-dipyrromethene (BODIPY) 581/591 C11 (Invitrogen; 2 μM) and quantified using the Em510 nm/Em590 nm ratio. After washing in phosphate-buffered saline (PBS), cells were analyzed with a FACSCantoII flow cytometer (BD Biosciences). Data were processed with FlowJo software (version 10.10.0; BD Biosciences).

### 2.5. Western Blot Analysis

FLSs were completely lysed using radioimmunoprecipitation assay (RIPA) buffer (ATTO, Co., Tokyo, Japan) containing protease and phosphatase inhibitors. Following centrifugation, supernatants were separated using sodium dodecyl sulfate–polyacrylamide gel electrophoresis (SDS–PAGE). Proteins were transferred onto polyvinylidene difluoride membranes (Bio-Rad Laboratories, Inc., Hercules, CA, USA) and blocked with 5% non-fat dry milk (BD Biosciences) in PBS with 0.05% Tween 20 (PBS-T). The membranes were incubated overnight at 4 °C with antibodies as indicated (Supplementary Data S1). All antibodies were diluted 1:1000 in PBS-T containing 5% non-fat dry milk. After washing with PBS-T, the membranes were stained with peroxidase-conjugated goat anti-rabbit IgG (AbFrontier Co., Seoul, Republic of Korea; 1:3000 dilution) or peroxidase-conjugated goat anti-mouse IgG (AbFrontier Co.; 1:3000 dilution). Chemiluminescent horseradish peroxidase (HRP) substrate (Thermo Fisher Scientific) was employed to visualize target proteins. The band intensities of these signals were quantified and compared with glyceraldehyde 3-phosphate dehydrogenase (GAPDH) or relative total protein using Total Lab TL120 (version 2.0.1; Nonlinear Dynamics, Newcastle upon Tyne, UK) (Supplementary Data S2).

### 2.6. Iron Quantification

An Iron Assay Kit (Cat# MAK025, Sigma-Aldrich, St. Louis, MO, USA) was used to measure ferrous and total iron levels. Cells were gently homogenized in 250 μL iron assay buffer and centrifuged at 15,900× *g* for 10 min at 4 °C. After removal of the insoluble pellet, the supernatant was used for iron assay following the manufacturer’s instructions. Levels were estimated by interpolation from a standard curve generated using a Sunrise absorbance reader (Tecan) at 593 nm. The iron concentration was calculated using the following formula.

Sa/Sv = C

Sa = Amount of iron in unknown sample from standard curve

Sv = Sample volume added into the well

C = Concentration of iron in sample

### 2.7. Glutathione Determination

Cells were stained for 30 min at 37 °C using an Intracellular GSH Detection Assay kit (Cat# ab112132, Abcam, Cambridge, UK) following the manufacturer’s instructions. The cells were then washed in PBS and analyzed using a FACSCantoII flow cytometer (BD Biosciences). Data were processed with FlowJo software (version 10.10.0; BD Biosciences).

### 2.8. Quantitative (q)RT-PCR

Following total RNA isolation using TRI Reagent (Molecular Research Center, Cincinnati, OH, USA), reverse transcription of RNA was performed with ReverTra Ace^®^ qPCR RT Master Mix (TOYOBO, Osaka, Japan) to generate first-strand cDNA according to the manufacturer’s instructions. SYBR^®^ Green Real-Time PCR Master Mix (TOYOBO) was employed for qRT-PCR analysis of the cDNA according to the manufacturer’s instructions. The primers were synthesized by Bioneer (Daejeon, Republic of Korea) and are listed in Supplementary Data S3 and S4. Thermal cycling was conducted on a CFX Connect Real-Time PCR Detection System (Bio-Rad Laboratories, Hercules, CA, USA). Target gene expression was quantified by calculation of the cycle threshold (Ct) values, with the results represented as a ratio relative to the level of *GAPDH* expression in the same sample. Relative expression levels of target genes were compared using the 2^−ΔΔCT^ method.

### 2.9. Statistical Analysis

Statistical analyses were performed using paired Student’s *t*-tests followed by Wilcoxon signed-rank tests in the SPSS 18.0 software (IBM, Armonk, NY, USA). Data are expressed as mean ± standard deviation (SD), median (min–max), or number and frequency. The threshold for statistical significance in all tests was *p* < 0.05.

## 3. Results

### 3.1. LKB1 Knockdown Increased Cell Death of RA FLSs

**Figure 1:**
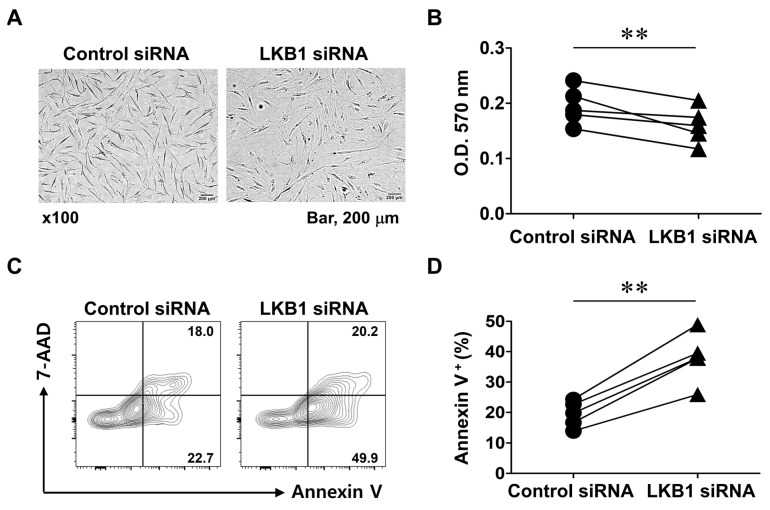
LKB1 deficiency results in increased death of rheumatoid arthritis fibroblast-like synoviocytes. RA FLSs were transfected with LKB1 siRNA or control siRNA. (**A**) Cells were photographed at 100× magnification 24 h after transfection. Data are from a representative experiment performed in triplicate. Scale bar, 200 μm. (**B**) Cell viability assessed by MTT assay 24 h after transfection. Pairs represent individual donors (*n* = 5). O.D., Optical density. (**C**,**D**) Cell death analyzed using FITC-conjugated Annexin V and 7-AAD staining 24 h after transfection. Data represent one median experiment (**C**) with percentages of Annexin V-positive populations shown for paired samples from each donor (*n* = 5) (**D**). ** *p*< 0.01.

LKB1 is regarded as an ROS scavenger, and LKB1 deficiency induces inflammation in RA FLSs [25]. To study the direct effect of LKB1 inhibition on cell survival, RA FLSs were transfected with LKB1-specific siRNA or control siRNA. Twenty-four hours after transfection, LKB1 mRNA levels were reduced by 0.12-fold in cells transfected with LKB1 siRNA compared with those in control cells. Cells transfected with control siRNA exhibited an elongated spindle morphology under light microscopy, without lipofectamine-induced damage (Figure 1A). In contrast, the LKB1-suppressed cells had markedly increased numbers of non-adherent cells that floated in the culture medium. MTT assays showed that the LKB1-suppressed cells (0.195 ± 0.033) had slightly reduced viability compared with the control cells (0.160 ± 0.032, *p* < 0.01) (Figure 1B). Annexin V staining showed that the percentage of apoptotic cells was increased when LKB1 was suppressed by 57.0% compared to control siRNA *(p* < 0.01) (Figure 1C,D). These findings suggest that LKB1 plays an important role in RA FLS cell survival.

### 3.2. LKB1 Regulates the Ferroptosis Pathway in RA FLSs

**Figure 2:**
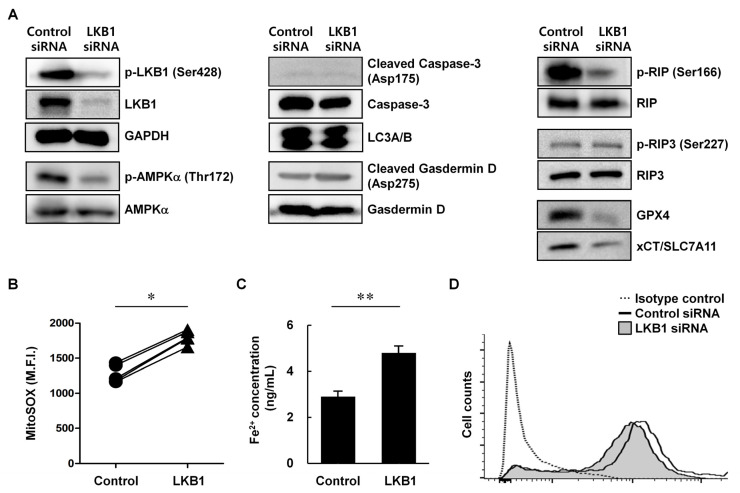
LKB1 deficiency-mediated cell death in rheumatoid arthritis fibroblast-like synoviocytes occurred through the ferroptosis pathway. RA FLSs were transfected with LKB1 siRNA or control siRNA. (**A**) Protein expression was analyzed by western blot 24 h after transfection. GAPDH was used as the loading control. (**B**–**D**) Mean fluorescence intensity (M.F.I.) of MitoSOX (**B**), ferrous (Fe^2+^) ion levels (**C**), and intracellular GSH expression (**D**) 24 h after transfection. Pairs in (**B**) represent individual donors (*n* = 5). Data presented as mean ± standard deviation from a representative experiment performed in triplicate. * *p* < 0.05. ** *p* < 0.01.

To determine the precise pathway of LKB1-dependent cell death, RA FLSs were transfected with LKB1 siRNA or control siRNA and used western blot to measure the levels of the autophagy marker LC3A/B and GPX4 and SLC7A11, which suppress the ferroptosis pathway and are used as markers of ferroptosis (Figure 2A). In addition, we measured the total and activated forms of LKB1, AMPKα (a downstream kinase that is directly bound and phosphorylated by LKB1 [27]), Caspase-3 (a marker of apoptosis), Gasdermin D (a marker of pyroptosis), and RIP and RIP3 (markers of necroptosis). The levels of total and phosphorylated (Ser428) LKB1 were markedly reduced in LKB1-suppressed cells compared with those in control cells. The total level of AMPKα was similar between the LKB1-suppressed cells and control cells, whereas the level of phosphorylated AMPKα (Thr172) was reduced in the LKB1-suppressed cells. There were no notable differences between the LKB1-suppressed cells and the control cells in the levels of cleaved Caspase-3 (Asp175), LC3A/B, cleaved Gasdermin D (Asp275), or phosphorylated RIP3 (Ser227). By contrast, the LKB1-suppressed cells displayed reduced levels of GPX4, SLC7A11, and phosphorylated RIP (Ser166).

Next, to examine ferroptosis in LKB1-suppressed RA FLSs directly, we measured the two major features of ferroptosis: lipid peroxide accumulation and ferrous ion overload. Compared with control cells, LKB1-suppressed RA FLSs displayed increased levels of mitochondria-specific ROS (by 70.8% compared to control siRNA, *p* < 0.05) and Fe^2+^ (by 60.4% compared to control siRNA, *p* < 0.01) (Figure 2B,C) and a decreased level of the antioxidant GSH (Figure 2D). Collectively, these results indicate that loss of LKB1 promotes cell death in RA FLSs by inducing activation of the ferroptosis pathway.

### 3.3. LKB1 Knockdown Increased the Sensitivity of RA FLSs to Ferroptosis

**Figure 3:**
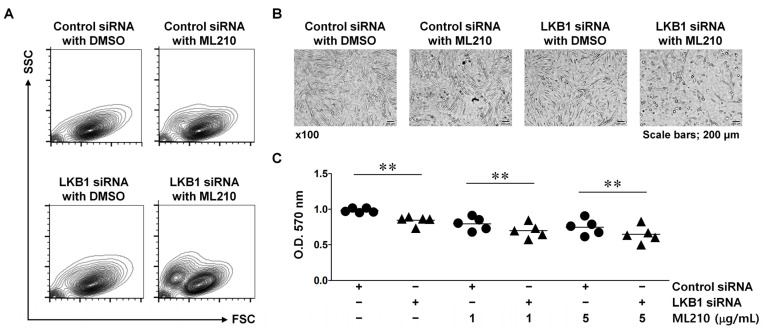
LKB1-deficient rheumatoid arthritis fibroblast-like synoviocytes are hypersensitive to ferroptosis-induced damage. RA FLSs were transfected with LKB1 siRNA or control siRNA. (**A**,**B**) Flow cytometry (**A**) and 100× microscopy (**B**) after stimulation with 1 μg/mL ML210 or dimethyl sulfoxide (DMSO) for 3 h. Scale bar, 200 μm. Data from a representative experiment performed in triplicate. (**C**) Viability after incubation with 1 or 5 μg/mL ML210 or DMSO for 24 h. Each symbol represents an individual donor (*n* = 5); ● means control siRNA and ▲ indicates LKB1 siRNA; bars represent means. O.D.; Optical density. ** *p* < 0.01.

To investigate the direct influence of LKB1 on ferroptosis sensitivity, LKB1-suppressed RA FLSs were stimulated with ML210, which can trigger ferroptosis by directly binding to GPX4 and causing lethal accumulation of membrane phospholipid hydroperoxides [28]. Cell damage was detectable by flow cytometry in control cells and LKB1-suppressed cells after treatment with ML210 for 3 h (Figure 3A). After treatment with 1 μg/mL or 5 μg/mL ML210 for 24 h, LKB1-suppressed cells displayed higher sensitivity to ML210-induced cell death compared with control cells (*p* < 0.01) (Figure 3B,C). These results suggest that LKB1 deficiency induces sensitivity to ferroptosis, resulting in irreversible damage and entry into the cell death pathway.

### 3.4. LKB1 Knockdown Increased Lipid Peroxidation in RA FLSs

**Figure 4:**
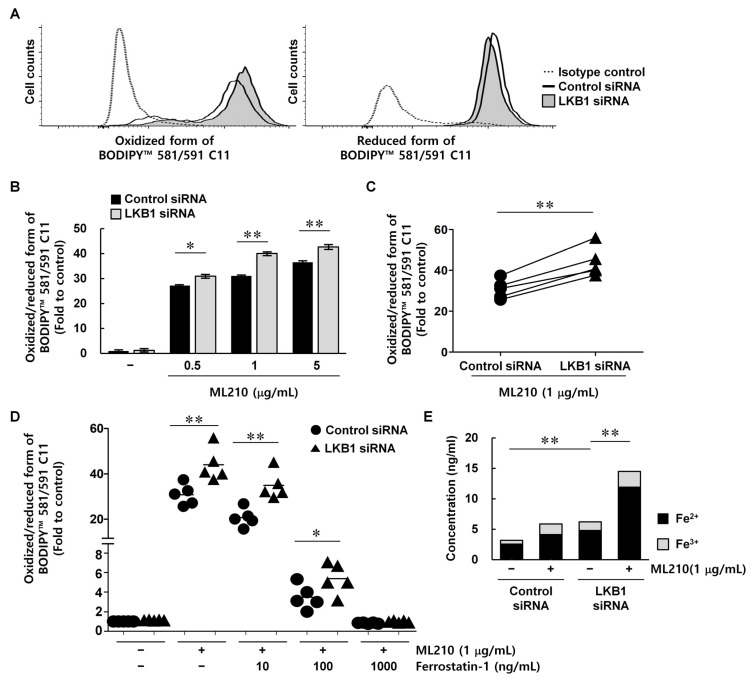
Generation of lipid reactive oxygen species is upregulated in LKB1-deficient rheumatoid arthritis fibroblast-like synoviocytes. RA FLSs were transfected with LKB1 siRNA or control siRNA. (**A**) Lipid peroxidation 24 h after transfection in a representative experiment performed in triplicate. (**B**,**C**) Membrane lipid peroxidation after incubation with 0.5, 1, or 5 μg/mL ML210 for 1 h. Data in (**B**) are mean ± standard deviation of one experiment conducted in triplicate with similar results. Pairs in (**C**) represent individual donors (*n* = 5). (**D**) Membrane lipid peroxidation after incubation with 1 μg/mL ML210 and 10, 100, or 1000 ng/mL ferrostatin-1 for 1 h. Symbols represent individual donors (*n* = 5); bars represent means. (**E**) Levels of ferrous (Fe^2+^) and ferric (Fe^3+^) ions after incubation with 1 μg/mL ML210 for 18 h. DMSO was used as the solvent control. Data represent one experiment conducted in triplicate with similar results. * *p* < 0.05. ** *p* < 0.01.

The main characteristic of ferroptosis is upregulation of lipid peroxidation, which can be measured using BODIPY lipid probes [29]. Because BODIPY dye shifts its emission fluorescence upon oxidation, the level of lipid peroxidation in membranes treated with BODIPY probes can be determined using the Em 510 nm/Em 590 nm ratio. When LKB1 was suppressed, the relative amount of oxidized probe in the cell membranes was increased and the amount of reduced probe was decreased (Figure 4A). Treatment of the cells with ML210 resulted in dose-dependent increases in the relative amount of oxidized probe, indicating increased lipid peroxidase levels, and the increase was greater in LKB1-suppressed cells than in control cells (Figure 4B). Pair-wise analysis of control and LKB1-suppressed RA FLSs from each patient showed that treatment with 1 μg/mL ML210 resulted in a greater increase of lipid peroxidation in LKB1-suppressed cells (44.01 ± 5.28) than in control cells (30.79 ± 4.65, *p* < 0.01) (Figure 4C). When RA FLSs were co-treated with 1 μg/mL ML210 and various doses of the ferroptosis inhibitor ferrostatin-1 [30], the ML210-induced increase in lipid peroxidation was reversed using ferrostatin-1 in a dose-dependent manner (Figure 4D). Furthermore, ferrostatin-1 induced even lower lipid peroxidation levels in LKB1-suppressed cells than in control cells. The ferrous ion levels were increased using ML210 treatment, and the extent of the increase was greater in LKB1-suppressed cells than in control cells (*p* < 0.01) (Figure 4E). These results suggest that LKB1 can induce ferroptosis susceptibility in RA FLSs.

### 3.5. LKB1 Suppression Was Restored by AMPK Activation in RA FLSs

**Figure 5:**
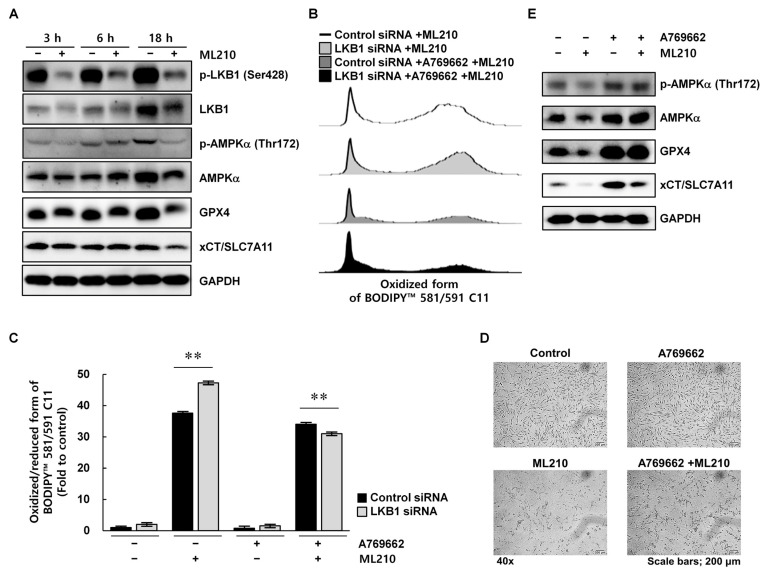
The AMPK activator A769962 restored LKB1 deficiency-mediated ferroptosis in rheumatoid arthritis fibroblast-like synoviocytes. (**A**) RA FLSs were stimulated with 1 μg/mL ML210 for 3, 6, or 18 h and analyzed using western blot with specific antibodies. (**B**,**C**) Cells were incubated with 250 μM A769662 for 1 h and then with 1 μg/mL ML210 for 1 h. Intracellular lipid peroxidation was analyzed using BODIPY probes. Data represent one median experiment conducted in triplicate with similar results. Data in (**C**) are presented as mean ± standard deviation. (**D**,**E**) Cells were incubated with 250 μM A769662 for 1 h and then with 1 μg/mL ML210 for 24 h (**D**) or 18 h (**E**). Scale bar, 200 μm. Cell lysates were analyzed using western blot with specific antibodies (**E**). DMSO and GAPDH were used as solvent and loading controls, respectively. ** *p* < 0.01.

To investigate whether ML210 affects LKB1-regulated ferroptosis signaling in RA FLSs, cells were treated with ML210 for 3, 6, or 18 h and measured the levels of GPX4, SLC7A11, and total and phosphorylated LKB1 and AMPKα (Figure 5A). The levels of phosphorylated LKB1 (Ser48) and AMPKα (Thr172) were markedly reduced in ML210-treated cells compared with those in solvent-treated control cells. The levels of GPX4 and SLC7A11 were also markedly reduced after ML210 stimulation. To determine whether LKB1 knockdown-mediated signal could be restored by AMPK activation, LKB1 siRNA-transfected RA FLSs were treated with A769662 to stimulate AMPKα-Thr172 phosphorylation and its downstream signaling [31]. ML210-induced lipid peroxidation was significantly reduced by pretreatment with the AMPKα activator and the reduction was greater in LKB1-suppressed cells than in control cells (*p* < 0.01) (Figure 5B,C). Cell viability was also markedly recovered by using A769662 pretreatment (Figure 5D). In RA FLSs co-stimulated with A769662 and ML210, the expression levels of GPX4 and SLC7A11 were increased compared with their expression in cells stimulated with ML210 alone (Figure 5E). Taken together, these results indicate that LKB1–AMPK signaling is essential for protecting RA FLSs from ferroptosis-mediated cell death.

## 4. Discussion

Most RA therapies focus on immune cells and their inflammatory responses [32]. However, these therapies attenuate disease activity, and cannot provide a fundamental cure. Therefore, new therapeutic options for patients with RA are needed. Because activated immune cells interact with FLSs and induce multiple cascades in the synovium, targeting RA FLSs holds promise as a treatment strategy that does not require systemic immune suppression [33]. Abnormal proliferation of FLSs in RA is usually accompanied by synovial hyperplasia and resistance to cell death, and ferroptosis induction in FLSs is an important research direction [34]. Administration of sulfasalazine, a common clinical treatment for RA, inhibits FLS proliferation, synovial inflammation, and joint swelling through ferroptosis induction in a collagen-induced arthritis (CIA) mouse model [35]. Therefore, targeting ferroptosis induction of FLSs could provide a resolution for synovial inflammation and is considered a novel therapeutic target for RA treatment. We previously reported that LKB1 silencing in RA FLSs induces mitochondrial ROS generation, an inflammatory response, and cell migration [25]. Because dysregulation of inflammation can cause cell disorder and death, in the present study, we investigated the direct effect of LKB1 on the viability of RA FLSs. LKB1 silencing in RA FLSs induced cellular damage and resulted in cell death (Figure 1). Previous studies showed that LKB1 regulates p53-dependent apoptosis [36] and nutrient limitation-induced autophagy [37]. LKB1-mediated AMPK activation is involved in chondrocyte pyroptosis [38] and results in the phosphorylation of RIPK1 in response to metabolic stress [39]. Although AMPK inactivation sensitizes cancer cells to ferroptotic cell death [40], no previous studies have focused on the role of LKB1 in relation to RA FLSs. We found that the survival reduction caused by LKB1 deficiency in RA FLSs was mainly due to activation of the ferroptosis pathway rather than the apoptosis, autophagy, pyroptosis, or necroptosis pathways (Figure 2). These findings suggest that LKB1 protects RA FLSs against ferroptotic cell death and is thus a key regulator of cell survival.

Transient suppression of LKB1 in RA FLSs induced reversible damage, but LKB1-suppressed cells could not be revived after ML210 treatment (Figure 3). Because LKB1 loss already initiates the ferroptosis pathway, ML210, which robustly induces ferroptosis, might differentially affect the sensitivity to ferroptosis in LKB1-suppressed cells and control cells. Ferroptosis is accompanied by the release of pro-inflammatory cytokines and is closely related to the inflammatory response [41]. During the ferroptotic process leading to cell death, multiple inflammatory-related signaling pathways are activated, promoting RA pathogenesis and temporarily aggravating the disease. LKB1 inhibition upregulated sensitivity to ferroptosis by inducing increases in lipid peroxidation and iron levels, which might lead to irreversible damage in RA FLSs (Figure 4). A previous study in the CIA mouse model found that RA drugs such as sulfasalazine alleviated synovial inflammation and joint swelling through ferroptosis-mediated pathway activation [35]. These findings suggest that FLS ferroptosis ultimately reduces the severity of synovitis in RA and might provide a novel avenue for the treatment of RA.

**Figure 6:**
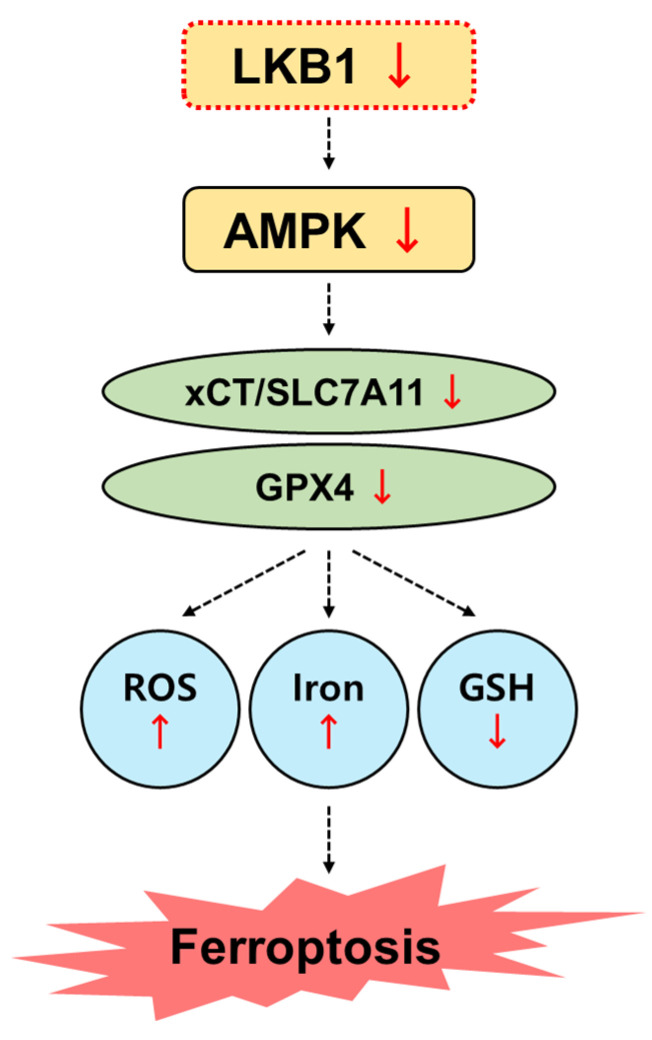
Schematic illustration of this study.

When the LKB1–AMPK axis was activated by AMPK in LKB1-suppressed cells, ML210-induced reductions in GPX4 and SLC7A11 expression were recovered, and ferroptosis sensitivity decreased (Figure 5). LKB1 plays important roles in regulating essential biological functions such as cellular metabolism, oxidative stress, and cell survival under glucose deprivation or hypoxia [42]. Ferroptosis is induced due to the accumulation of ROS or polyunsaturated fatty acids, which are closely related to energy suppliers and signaling molecules [43]. Therefore, targeting the LKB1-mediated ferroptosis pathway is considered a promising therapeutic approach for conditions of oxidative stress and lipid/iron metabolism-related disease (Figure 6). However, more direct and precise evidence is needed to verify the connection of these pathways in ferroptosis. Notably, this study was performed using ex vivo human FLSs; these effects do not fully translate well to in vivo systems. Further studies are needed in RA animal models to evaluate the therapeutic potential of targeting LKB1. Despite this limitation, our findings provide new evidence highlighting the role of LKB1-regulated ferroptosis in RA pathology.

## 5. Conclusions

The results of this study suggest that LKB1 protects RA FLSs against ferroptotic cell death and is thus a key regulator of cell survival. Future studies employing animal models of RA are warranted to better understand the role of LKB1 in this disease context.

## Figures and Tables

**Table 1:** Baseline characteristics of all patients included in this study.

Variables		Subjects with Knee RA (*n* = 5)
Female (*n*, %)		4 (80)
Age (year, mean ± SD)		53.2 ± 11.1 (35–61)
Duration of disease (month, mean ± SD)		143.6 ± 79.3 (40–240)
Rheumatoid factor–positive, *n* (%)		5 (100)
Anti CCP antibody–positive, *n* (%)		4 (80)
DAS28 (ESR, mean ± SD)		2.84 ± 1.72 (1.75–5.81)
Duration of treatment (month, mean ± SD)		141 ± 85 (28–240)
Treatment (*n*, %)	Naïve	0
	Steroid	4 (80)
	Methotrexate	4 (80)
	Hydroxychloroquine	3 (60)
	Sulfasalazine	1 (20)
	Leflunomide	3 (60)
	Tacrolimus	1 (20)
	Biologic DMARD	1 (20), Golimumab

SD: standard deviation; CCP: cyclic citrullinated peptide; DAS28: disease activity score of 28 joint counts; DMARD: disease-modifying antirheumatic drug.

## Data Availability

The data presented in this study are available on request from the corresponding author due to privacy or ethical restrictions.

## Supplementary Materials

The following supporting information can be downloaded at: https://www.mdpi.com/article/10.3390/biomedicines13020321/s1, Supplementary Data S1: Key resources; Supplementary Data S2: Relative quantification of selected proteins from western blot analysis; Supplementary Data S3: Primers used for PCR; Supplementary Data S4: Uncropped images from Western blots.

## Abbreviations

The following abbreviations are used in this manuscript:

AMPK	AMP-activated protein kinase
BODIPY	boron-dipyrromethene
CIA	collagen-induced arthritis
FLSs	fibroblast-like synoviocytes
GSH	glutathione
GPX4	GSH peroxidase 4
IL	interleukin
LC3	light chain 3
LKB1	Liver kinase B1
NADPH	nicotinamide adenine dinucleotide phosphate
NOX4	NADPH oxidase 4
RA	rheumatoid arthritis
RIPK	receptor-interacting protein kinase
ROS	reactive oxygen species
SLC7A11	solute carrier family 7 member 11
TNF	tumor necrosis factor
