# Supplementary material for: The LKB1–AMPK Signaling Axis Modulates Ferroptosis in Fibroblast-Like Synoviocytes Derived from Rheumatoid Arthritis
Source: Biomedicines. 2025 Jan 30;13(2):321. doi: 10.3390/biomedicines13020321 (PMC11853117; doi:10.3390/biomedicines13020321)
Supplement: Supplementary file 1 [file biomedicines-13-00321-s001.zip › biomedicines-3445829-supplementary.pdf]

Supplementary Data S1. Key resources

Name	Company	Product number	Dilution factor
Antibodies			
Anti-human phospho-LKB1 (Ser428)	Cell Signaling Technology	3482	1:1000
Anti-human LKB1	Cell Signaling Technology	3047	1:1000
Anti-human phospho-AMPKα (Thr172)	Cell Signaling Technology	2531	1:1000
Anti-human AMPKα	Cell Signaling Technology	2532	1:1000
Anti-human cleaved Caspase-3 (Asp175)	Cell Signaling Technology	9664	1:1000
Anti-human Caspase-3	Cell Signaling Technology	14220	1:1000
Anti-human LC3A/B	Cell Signaling Technology	12741	1:1000
Anti-human cleaved Gasdermin D (Asp275)	Cell Signaling Technology	36425	1:1000
Anti-human Gasdermin D	Cell Signaling Technology	97558	1:1000
Anti-human phospho-RIP (Ser166)	Cell Signaling Technology	65746	1:1000
Anti-human RIP	Cell Signaling Technology	73271	1:1000
Anti-human phospho-RIP3 (Ser227)	Abcam	ab209384	1:1000
Anti-human RIP3	Santa Cruz Biotechnology	sc374639	1:1000
Anti-human GPX4	Cell Signaling Technology	52455	1:1000
Anti-human xCT/SLC7A11	Cell Signaling Technology	12691	1:1000
Anti-human GAPDH	Cell Signaling Technology	2118	1:1000
Assay kit and chemicals			
Cell Proliferation Kit I (MTT)	Roche	11465007001	
Iron Assay Kit	Sigma-Aldrich	MAK025	
Intracellular glutathione (GSH) Detection Assay Kit	Abcam	ab112132	
FITC-conjugated Annexin V	BD Biosciences	556420	1:100
7-Amino-Actinomycin (7-AAD)	BD Biosciences	559925	1:20
MitoSOX™ Mitochondrial Superoxide Indicators	Invitrogen	M36008	
BODIPY™ 581/591 C11 (Lipid Peroxidation Sensor)	Invitrogen	D3861	
ML210	Sigma-Aldrich	SML0521	
Ferrostatin-1	Sigma-Aldrich	SML0583	
A769662	Sigma-Aldrich	SML2578	

Cell Signaling Technology (Danvers, MA, USA), Abcam (Cambridge, UK), Santa Cruz Biotechnology (Dallas, TX, USA), Roche (Basel, Switzerland), Sigma-Aldrich (St. Louis, MO, USA), BD Biosciences (Franklin Lakes, NJ, USA), Invitrogen (Carlsbad, CA, USA).

Supplementary Data S2. Relative quantification of selected proteins from western

blot analysis

The band intensities of Figure 2A (**A**), Figure 5A (**B**) and Figure 5E (**C**) were quantified and compared with GAPDH or relative total protein using Total Lab TL120 (version 2.0.1; Nonlinear Dynamics, Newcastle upon Tyne, UK).

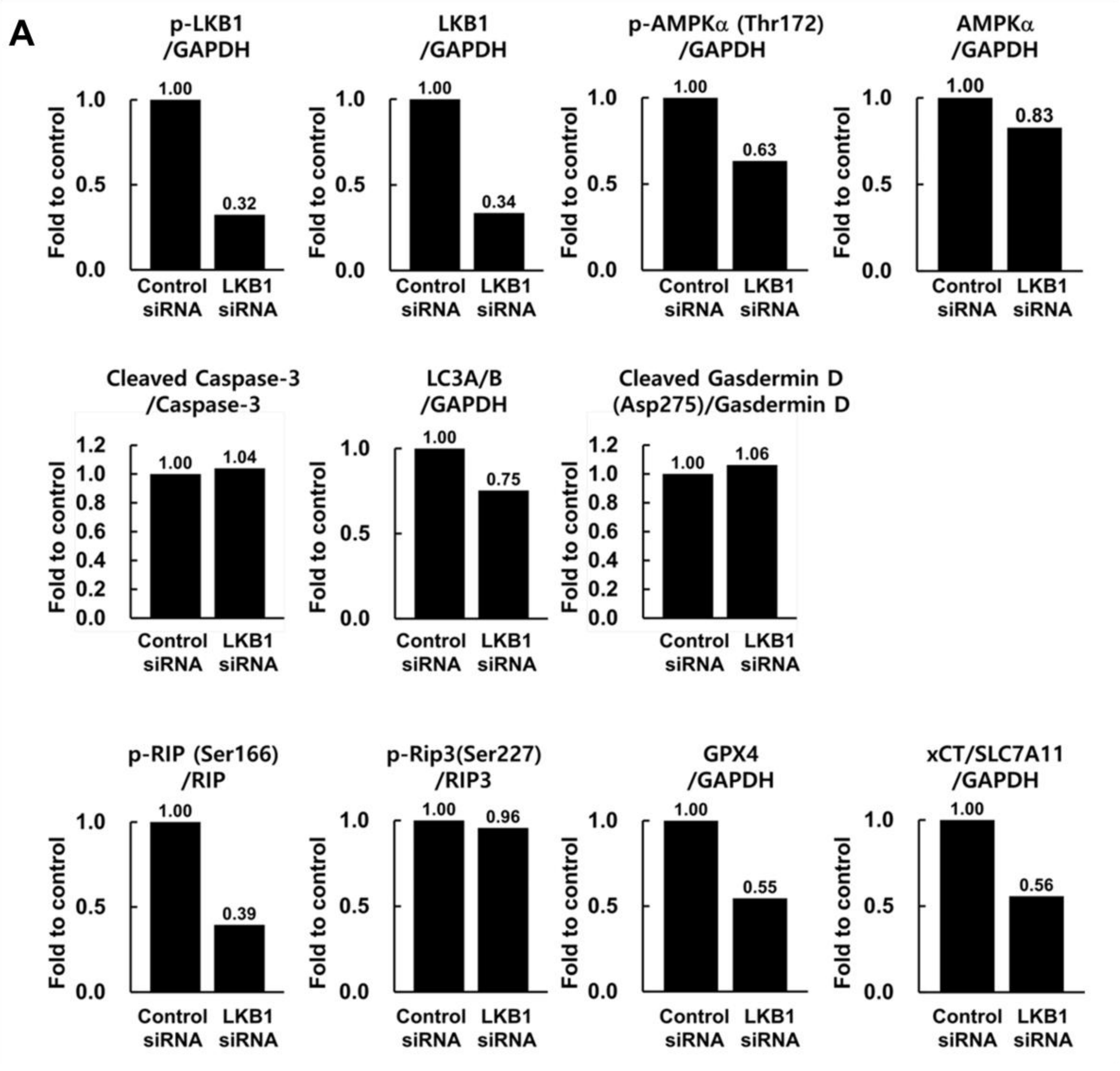

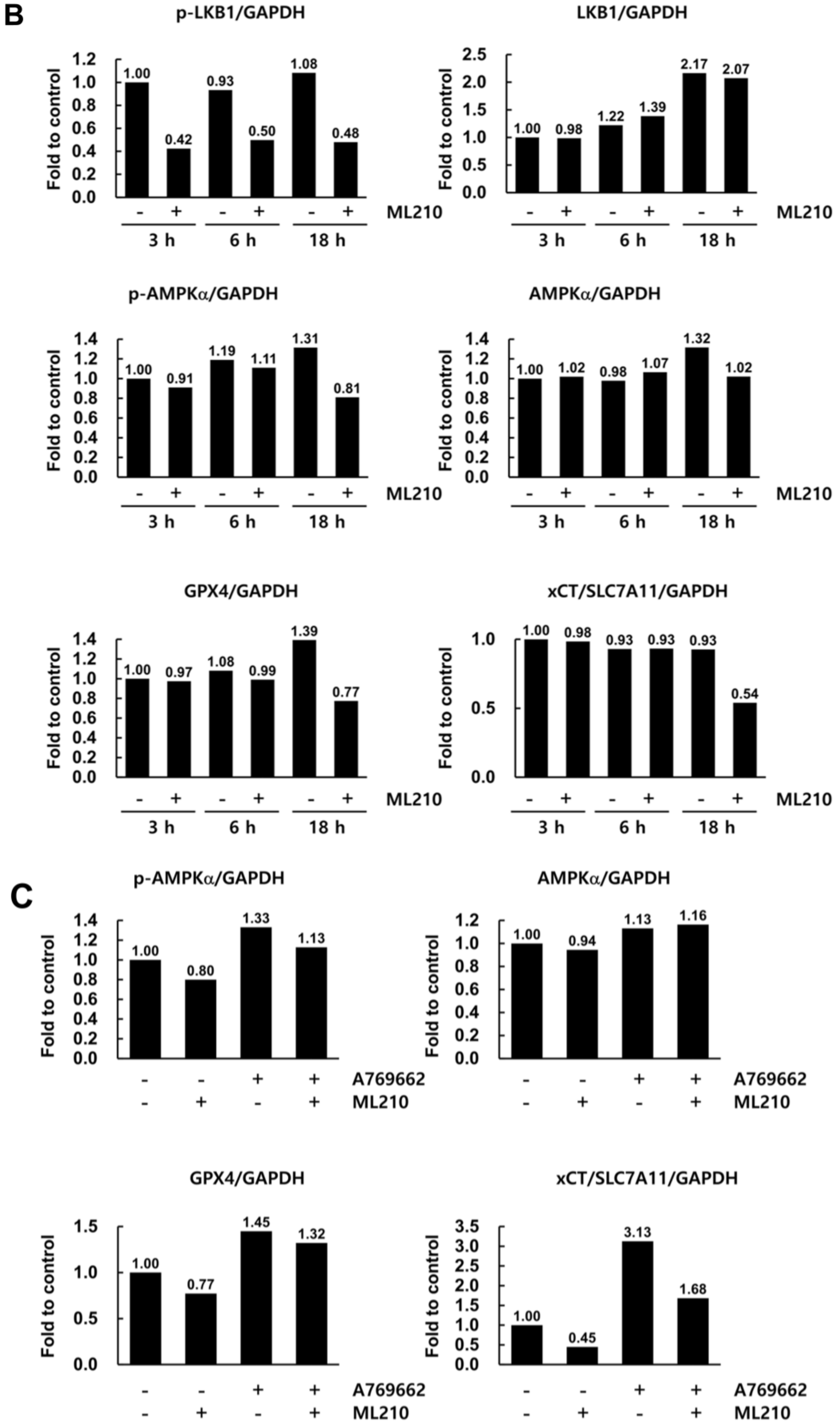
B
p-LKB1/GAPDH
Fold to control
1.00
0.42
0.93
0.50
1.08
0.48
ML210
3 h
6 h
18 h
LKB1/GAPDH
1.00
0.98
1.22
1.39
2.17
2.07
p-AMPKα/GAPDH
1.00
0.91
1.19
1.11
1.31
0.81
AMPKα/GAPDH
1.00
1.02
0.98
1.07
1.32
1.02
GPX4/GAPDH
1.00
0.97
1.08
0.99
1.39
0.77
xCT/SLC7A11/GAPDH
1.00
0.98
0.93
0.93
0.93
0.54
C
p-AMPKα/GAPDH
1.00
0.80
1.33
1.13
A769662
ML210
AMPKα/GAPDH
1.00
0.94
1.13
1.16
GPX4/GAPDH
1.00
0.77
1.45
1.32
xCT/SLC7A11/GAPDH
1.00
0.45
3.13
1.68

Supplementary Data S3. Primers used for PCR

	Sense primer (5’ to 3’)	Antisense primer (5’ to 3’)
IL-6	AACCTGAACCTTCCAAAGATGG	TCTGGCTTGTTCCTCACTACT
IL-8	CATACTCCAAACCTTTCCACCCC	TCAGCCCTCTTCAAAAACTTCTCCA
TNF-α	CCCGAGTGACAAGCCTGTAG	GATGGCAGAGAGGAGGTTGAC
VEGF	TCTTGGGTGCATTGGAGCCTC	AGCTCATCTCTCCTATGTGC
GAPDH	CACATGGCCTCCAAGGAGTAA	TGAGGGTCTCTCTCTTCCTCTTGT

Supplementary Data S4. Uncropped images from Western blots

Uncropped Western blot images are shown that correspond to Figure 2A (**A**), Figure 5A (**B**), and Figure 5E (**C**).

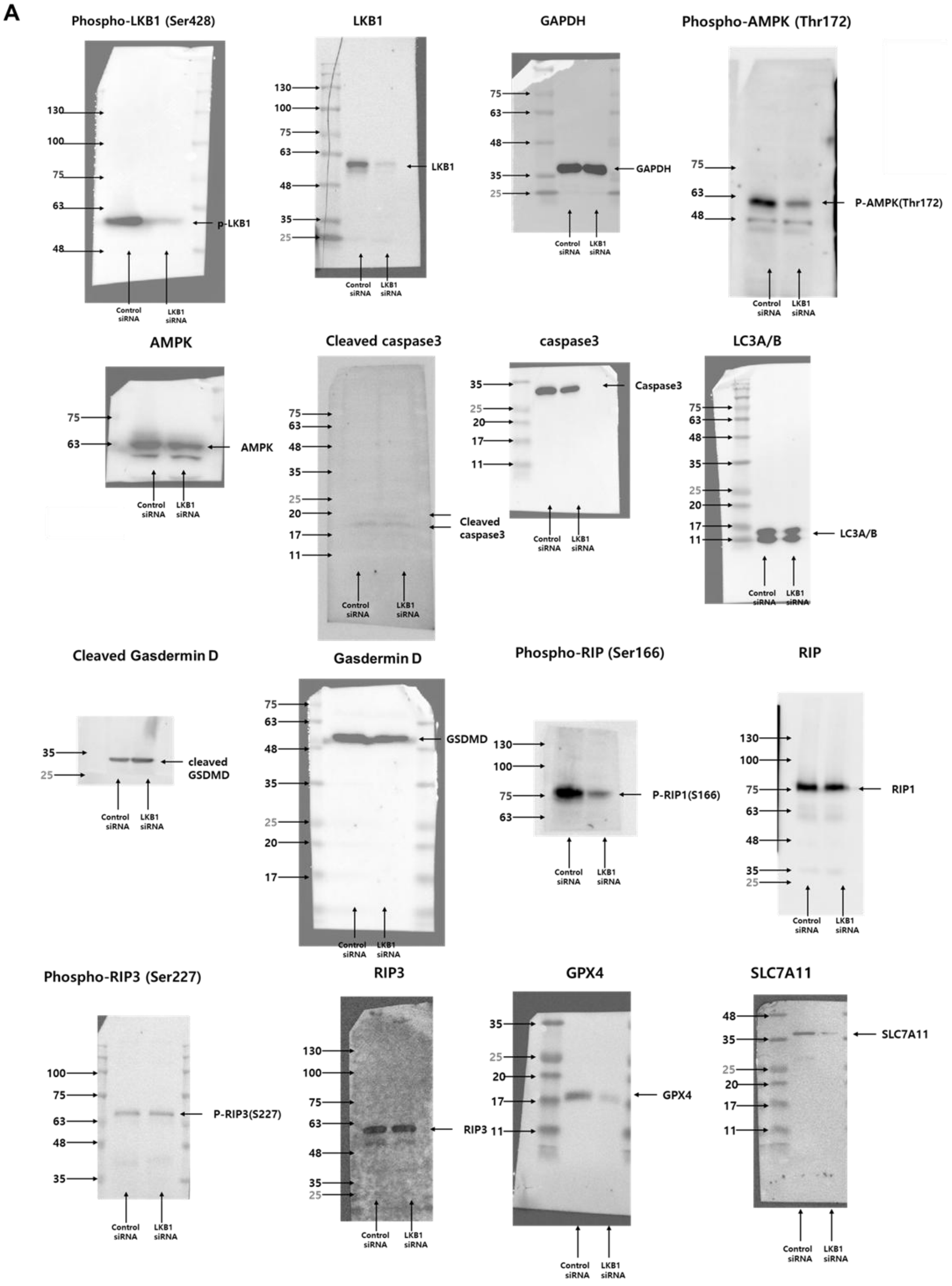

B

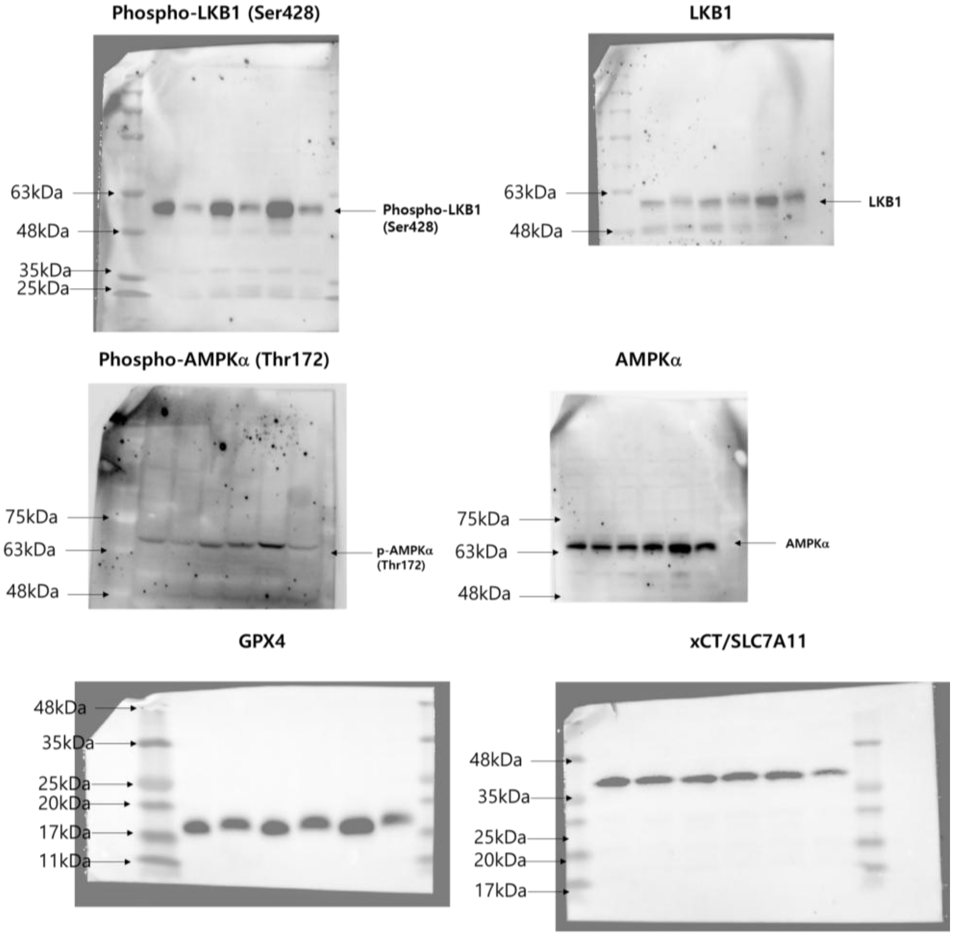
Phospho-LKB1 (Ser428)
63kDa
48kDa
35kDa
25kDa
Phospho-LKB1 (Ser428)
LKB1
63kDa
48kDa
LKB1
Phospho-AMPKα (Thr172)
75kDa
63kDa
48kDa
p-AMPKα (Thr172)
AMPKα
75kDa
63kDa
48kDa
AMPKα
GPX4
48kDa
35kDa
25kDa
20kDa
17kDa
11kDa
xCT/SLC7A11
48kDa
35kDa
25kDa
20kDa
17kDa

C

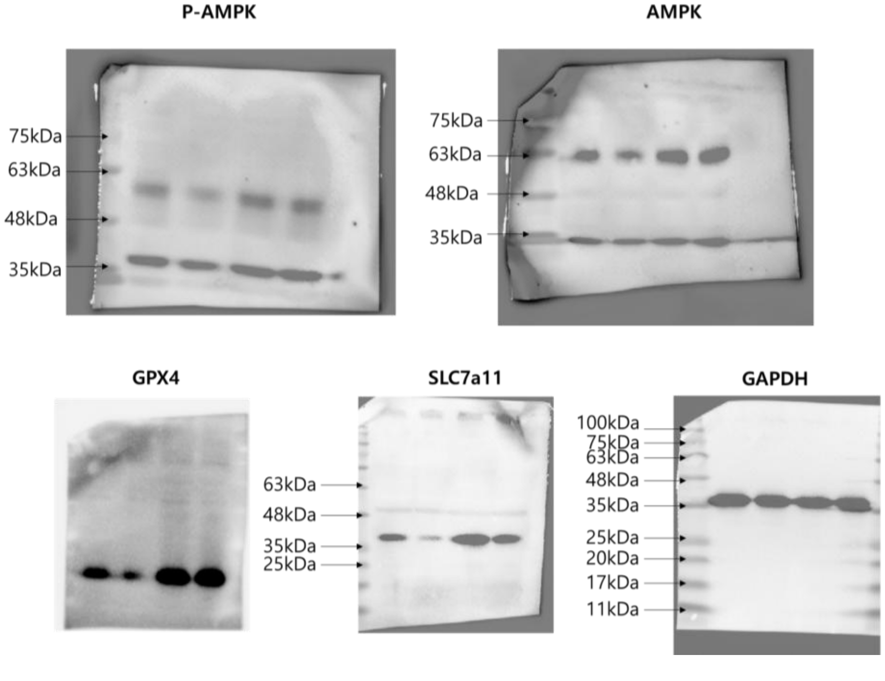
P-AMPK
75kDa
63kDa
48kDa
35kDa
AMPK
75kDa
63kDa
48kDa
35kDa
GPX4
SLC7a11
63kDa
48kDa
35kDa
25kDa
GAPDH
100kDa
75kDa
63kDa
48kDa
35kDa
25kDa
20kDa
17kDa
11kDa
